# On-Chip Structures for *F_max_* Binning and Optimization †

**DOI:** 10.3390/s22041382

**Published:** 2022-02-11

**Authors:** Dongrong Zhang, Qiang Ren, Donglin Su

**Affiliations:** School of Electronic and Information Engineering, Beihang University, Beijing 100191, China; dongrongzhang@buaa.edu.cn (D.Z.); sdl@buaa.edu.cn (D.S.)

**Keywords:** on-chip sensor, speed binning, yield optimization, dynamic adaptation

## Abstract

Process variations during manufacturing lead to differences in the performance of the chips. In order to better utilize the performance of the chips, it is necessary to perform maximum operation frequency ($F_{max}$) tests to place the chips into different speed bins. For most $F_{max}$ tests, significant efforts are put in place to reduce test cost and improve binning accuracy; e.g., our conference paper published in ICICM 2017 presents a novel binning sensor for low-cost and accurate speed binning. However, by promoting chips placed at the lower bins, because of conservative binning, into higher bins, the overall profit can greatly increase. Therefore, this paper, extended based on a conference paper, presents a novel and adaptive methodology for speed binning, in which the paths impacting the speed bin of a specific IC are identified and adapted by our proposed on-chip *Binning Checker* and *Binning Adaptor*. As a result, some parts at a bin margin can be promoted to higher bins. The proposed methodology can be used to optimize the $F_{max}$ yield of a digital circuit when it has redundant timing in clock tree, and it can be integrated into current $F_{max}$ tests with low extra cost. The proposed adaptive system has been implemented and validated on five benchmarks from ITC, ISCAS89, and OpenSPARCT2 core on 28 nm Altera FPGAs. Measurement results show that the number of higher bin chips is improved by 7–16%, and our cost analysis shows that the profit increase is between 1.18% and 3.04%.

## 1. Introduction

Process variation during manufacturing impacts the oxide thickness, threshold voltage, etc. of the transistor in integrated circuits, resulting in path delay fluctuations [1,2]. Due to these fluctuations, the performance of chips is different; hence, chips (microprocessors, DSPs, micro controllers, and sometimes even ASICs) can be placed into different speed bins [3,4]. Chips with higher performance are placed into higher speed bins, which can bring more profit. For instance, the price of the fastest Intel Prescott^TM^ and AMD64 Venice^TM^ device is about three times higher than that of the slowest parts [5]. To obtain more profit, it is necessary to promote the proportion of faster chips. Hence, efficiently and accurately $F_{max}$ tests are required to prevent high-performance chips from being placed into low bins. Another approach is to make efforts to promote low-end chips to higher bins and improve the proportion of high-end chips.

Generally, speed binning can be achieved by performing at-speed $F_{max}$ tests [6], which can be divided into functional, structural (scan-based), and sensor-based tests. The functional $F_{max}$ test is to find the maximum operation frequency at which the chip can operate normally by applying test patterns at different clock frequencies in the functional mode [7]. This requires the use of high-end automated test equipment (ATE) to apply and analyze a large number of test patterns at high speed, resulting in high test overhead. In order to reduce the cost of testing, part of the work adopts a Software-Based Self-Test (SBST) [8,9,10] to perform on-chip storing and analyzing of test patterns, which reduces the requirements for high-end ATE. SBST usually requires large on-chip memory storage.

The structural (scan-based) $F_{max}$ test includes LOC (launch on shift), LOS (launch on capture), and LOES (launch on extra shift) tests [11,12,13]. During the structural $F_{max}$ test, at-speed test patterns are shifted through scan chains with a low speed scan clock, and then, the test is performed with one or two high-speed functional clock cycles [14,15,16]. The test responses are scanned out for analyzing to get the actual speed bins. [17] investigates the correlation between functional test frequency and that of structural test patterns. In [18], a formula relating structural critical path testing frequency to system operation frequency is offered, which shows that a structural test can be employed to reduce the speed binning dependency on functional tests. Using the on-chip programmable PLL circuitry to obtain high-frequency clocks for an at-speed scan test is presented in [19]. Ref. [20] focuses on generating high-quality structural binning patterns considering the impact of process variations and proposes a new pattern-generation methodology for speed binning.

Generally, the speed bin of a chip is determined by the delay of critical paths. Hence, on-chip sensors, which can measure the delay of the critical paths [21,22,23,24,25,26], or monitor the worst slack of critical paths [27,28,29], are adopted to infer the speed bins of the chip. A low-overhead solution for characterizing the $F_{max}$ of a circuit is proposed in [30], which chooses a small set of representative paths in a circuit and dynamically configures them into ring oscillators to compute the $F_{max}$.Sensors combined with machine learning [31,32,33] and data mining are also applied to the speed binning test. Refs. [34,35] predict $F_{max}$ through data mining of the measured on-chip performance monitors. In addition, for higher profit, a methodology is proposed in [36,37] to adjust the bin boundaries according to the test result. An $F_{max}$ test based on on-chip sensors has a lower requirement for high-end external equipment than a functional test [38], and it takes less time than a structural test. This type of test has gradually become popular in recent years.

Due to process variations and noise, a path fails at a binning frequency with a certain statistical probability. At the same time, the failing paths are different from one chip to another. Therefore, to increase the $F_{max}$ yield (here, $F_{max}$ yield is defined as minimizing misplacing chips at lower bins and increasing the number of chips placed at higher bins), it is important to accurately identify and adapt the failing paths at a binning frequency. Hence, in this paper, a novel on-chip adaptive speed-binning system for $F_{max}$ yield optimization is proposed, which can be used to optimize the $F_{max}$ yield of a digital circuit when it has redundant timing in clock tree, the advantages of which are summarized below:

- Based on the $F_{max}$ test results, the proposed system can improve the $F_{max}$ yield and increase the overall profit by promoting chips from lower speed bins to higher speed bins.
- The proposed system can work seamlessly with existing $F_{max}$ tests, including functional, structural, and sensor-based tests.
- The proposed on-chip adaptive speed-binning system is all digital with negligible area and test overhead.

It should be noted that this paper is an extended version of our paper published in the 2017 2nd IEEE International Conference on Integrated Circuits and Microsystems (ICICM) [29]. The ICICM 2017 paper [29] only focuses on a novel binning sensor for low-cost and accurate speed binning, while this paper focuses on promoting chips placed in the lower bins into higher bins. This paper improves the binning sensor architecture in the ICICM paper, and it proposes a novel on-chip adaptation binning and $F_{max}$ yield optimization system. Moreover, it presents a novel and adaptive methodology for yield optimization, in which the paths impacting the speed bin of a specific IC are identified and adapted by our proposed on-chip *Binning Checker* and *Binning Adaptor*. As a result, some ICs placed in the lower bins can be promoted into higher bins. Hence, the overall profit can be increased. Due to the different design purposes and test method, the two papers provide different experimental results. In summary, there is about a 70–80% difference between the ICICM paper [29] and this paper.

The rest of the paper is organized as follows. The architecture of the proposed system is described in Section 2. The system implementation and $F_{max}$ yield optimization flow is presented in Section 3. Section 4 shows the experimental and measurement results. Finally, Section 5 gives the concluding remarks.

## 2. Architecture

**Figure 1:**
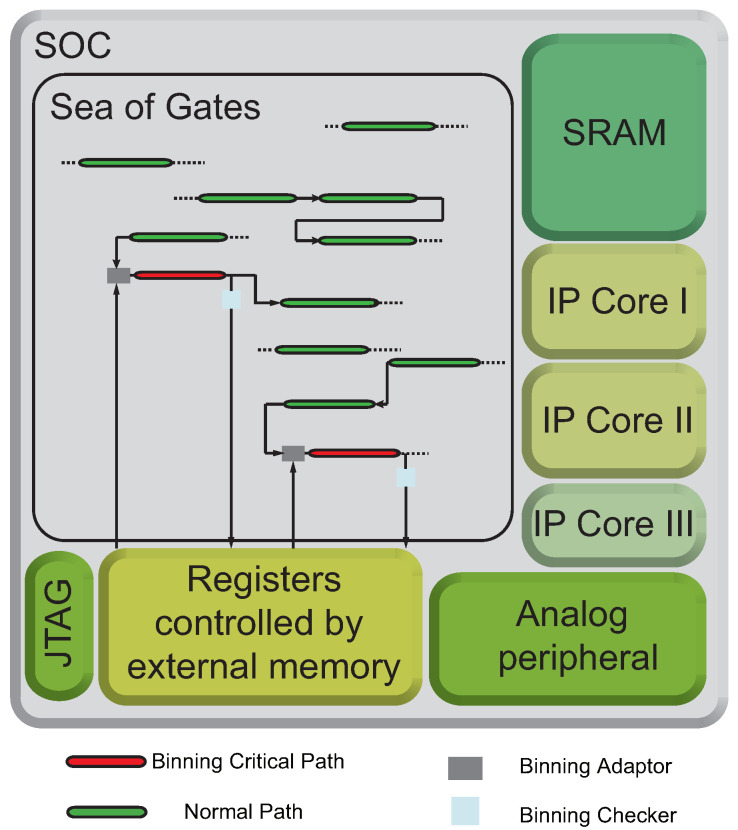
Overview of the proposed on-chip adaptive binning and $F_{max}$ yield optimization system.

The proposed on-chip adaptive binning and $F_{max}$ yield optimization system is composed of *Binning Checkers*, *Binning Adaptors*, and some on-chip flash memory, as shown in Figure 1.

### 2.1. The Binning Checker

**Figure 2:**
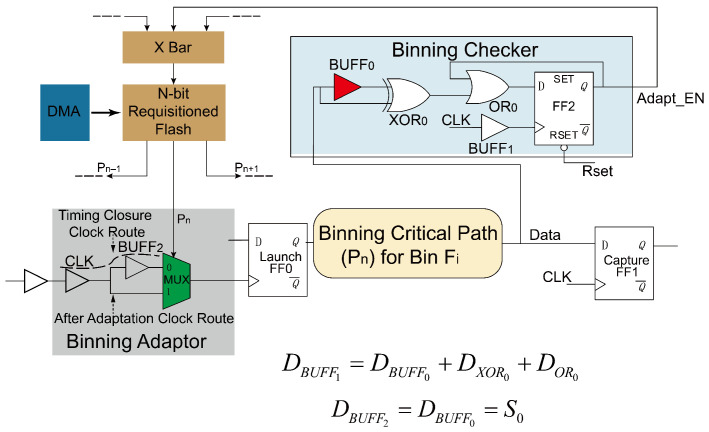
The *Binning Checker* and *Binning Adaptor*.

Generally, the speed bin of a chip is determined by some of the longest paths, which are named as *Binning Critical Paths*. The *Binning Critical Path* selection methodology is presented in detail in Section 4.3. Due to process variations and noise, a *Binning Critical Path*’s delay may exceed the bin boundary, which means the output of the path switches after the capture clock. Therefore, the capture flip-flop obtain obtains wrong data, making the chip drops drop to a lower bin during response analysis. The proposed *Binning Checker* utilizes this feature to monitor whether the timing critical path’s delay is longer than the applied clock frequency. The detailed structure of the *Binning Checker* is shown in Figure 2. As the *Binning Checker* only includes a few standard gates, a dedicated *Binning Checker* can be attach attached at the end of each *Binning Critical Path*. The *Binning Checker* has two tasks:

- Task 1: It locates the paths causing the $F_{i}$ binning failure on silicon, where $F_{i}$ is the frequency boundary between $bin_{i}$ and its higher bin $bin_{i-1}$.
- Task 2: It evaluates whether the located *Binning Critical Paths* can be adapted to $bin_{i-1}$ by the proposed *Binning Adaptor*.

If both (1) and (2) are satisfied, the output of *Binning Checker* (Adapt_EN), as shown in Figure 2, is switched to *1*, which initiates the adaptive binning and $F_{max}$ yield optimization.

As shown in Figure 2, the *Binning Checker* is used to monitor the output of *Binning Critical Path*. The clock frequency of the critical path is the binning frequency $F_{i}$. Assume the adaptable margin is $S_{0}$, which is equal to the delay of $BUFF_{0}$ inside the *Binning Checker*. To achieve Task 1, the two inputs of the $XOR_{0}$ gate come from the output of the critical path (node Data in Figure 2) and the critical path output signal through the $BUFF_{0}$, respectively. Thus, the $XOR_{0}$ gate outputs *1* if the data transit from *0* to *1* or *1* to *0* within $S_{0}$. The $OR_{0}$ gate and FF2 together form a “sticky” structure, which means that once FF2 outputs *1*, the value of FF2 remains *1* until it is reset. Before binning optimization, all FF2s in *Binning Checker* are reset. $BUFF_{1}$ is composed by several buffer cells, and its delay is equal to the sum of the delay of $BUFF_{0}$, $XOR_{0}$, and $OR_{0}$, as shown in Figure 2. Hence, FF2 can monitor data transition after the capture edge of CLK within $S_{0}$. If the Data (the output of *Binning Critical Path*) transition occurs within $S_{0}$ after the clock capturing, the output of *Binning Checker* (Adapt_EN) would turn to *1*. Adapt_EN=1 means that (i) the delay of the *Binning Critical Path* is greater than $1/F_{i}$, and (ii) the delay of *Binning Critical Path* is smaller than $1/F_{i}+S_{0}$. To make the detected failing path recoverable, $BUFF_{0}$ inside the *Binning Checker* needs to be the same as $BUFF_{2}$ inside the *Binning Adaptor* (Figure 2). The *Binning Adaptor* is introduced in detail in Section 2.2. By employing the design above, Task 1 and 2 mentioned above are fully achieved.

**Figure 3:**
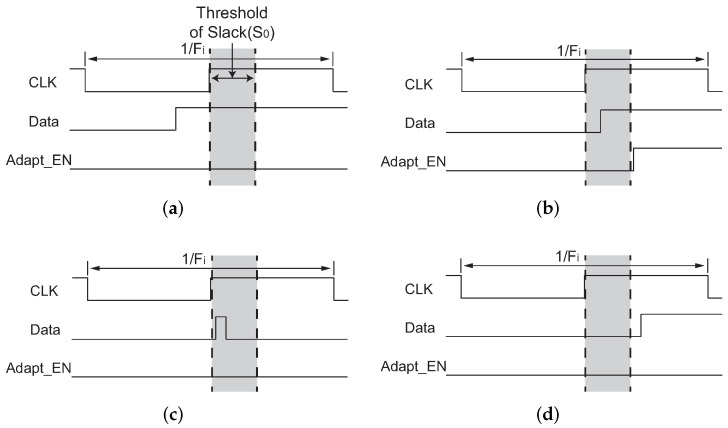
Four conditions of data/clock transition at $FF_{1}$($FF_{2}$), and the adaptation decision made by the *Binning Checker*. (**a**) The monitored path does not cause binning failure, *Adapt_EN* stays at *0*; (**b**) The monitored path is an adaptable *Binning Critical Path*, *Adapt_EN* switches to *1*; (**c**) Glitch exists within $S_{0}$ after capturing clock. *Adapt_EN* stays at *0* to avoid misjudgement and wrong adaptation; (**d**) The monitored path is an unadaptable *Binning Critical Path*. *Adapt_EN* stays at *0*.

Figure 3 shows the output of Adapt_EN under different Data transition conditions. In Figure 3a, the variable Data turns to *1* before clock capturing, which means that the monitored path does not cause binning failure at $F_{i}$. Hence, the output of Adapt_EN stays at *0*. In Figure 3b, the variable Data turns to *1* within $S_{0}$ after clock capturing, which means that the monitored path is an adaptable *Binning Critical Path*. Thus, the output of Adapt_EN turns to *1* to initiate adaptive binning. In Figure 3c, there is a glitch in $S_{0}$ after capturing. Then, the output of Adapt_EN stays at *0* to avoid misjudgement and wrong adaptation. In Figure 3d, the moment that the variable Data turns to *1* is out of adaptable range, which means that the monitored path is an unadaptable *Binning Critical Path*. Thus, the output of Adapt_EN stays at *0*.

### 2.2. The Binning Adaptor

The *Binning Adaptor* is designed to recover the identified adaptable failure *Binning Critical Paths*. The circuit for *Binning Adaptor* is also shown in Figure 2. The *Binning Adaptors* are inserted into the selected *Binning Critical Paths*, which moves the launching clock ahead to achieve a longer clock cycle. In other words, the *Binning Adaptor* can borrow a redundant margin from its upper stream path when necessary. Gate $MUX_{0}$ of the *Binning Adaptor* is inserted at the end of the original clock network for $FF_{0}$. To make the *Binning Adaptor* insertion have little impact on the already closed timing, a number of buffers in the original clock tree need to be removed to cancel the effect of MUX insertion. From Figure 2, it can be seen that there are two configurable routes for the clock (CLK) going through the *Binning Adaptor*, namely the *Timing Closure Clock Route* and the *After Adaptation Clock Route*. Clearly, the clock cycle of *After Adaptation Clock Route* is $S_{0}$ longer than the *Timing Closure Clock Route*. The $MUX_{0}$ is controlled by the *Binning Checker* inserted into the same path. When the adaptation decision is made by the *Binning Adaptor*, the *Binning Checker* switches the route of launch clock to *After Adaptation Clock Route*. Thus, the failed adaptable *Binning Critical Path* can be recovered. Then, Adapt_EN is written into a directly accessible flash memory to guarantee the function of the path for the later powering ups.

**Figure 4:**
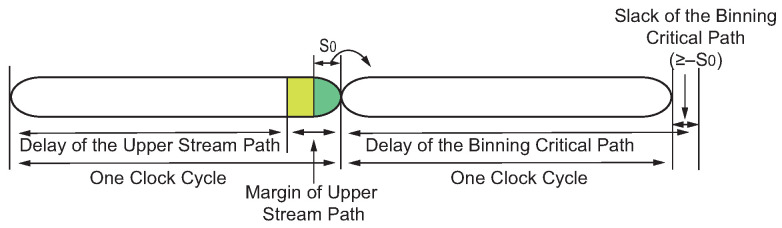
*Binning Adaptor* borrows unused timing margin from the upper stream path to an adaptable *Binning Critical Path*.

**Figure 5:**
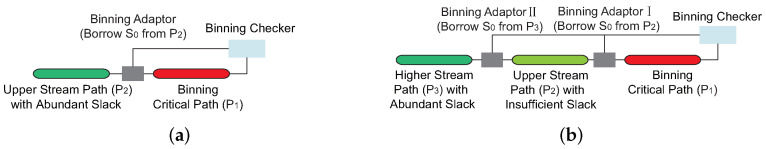
The insertion of *Binning Adaptor* under two different scenarios. (**a**) The upper stream path of the *Binning Critical Path* has a slack larger than $S_{0}$. Then only one Binning Adaptor is needed; (**b**) The upper stream path of the *Binning Critical Path* has a slack smaller than $S_{0}$. Hence multiple Binning Adaptors are inserted.

Figure 4 shows the timing of a *Binning Critical Path* and the margin borrowed from its upper stream path. To make the margin borrowing possible, the upper stream path should still function after lending margin $S_{0}$ to the failed *Binning Critical Path*. Hence, the upper stream path should have a margin larger than $S_{0}$. Therefore, the upper stream path and *Binning Critical Path* could both support binning boundary frequency $F_{i}$. It should be noted that when the upper stream path has sufficient margin, only one *Binning Adaptor* is needed to be inserted, as shown in Figure 5a. However, sometimes, the timing margin of the upper stream path does not meet this requirement. Then, multiple *Binning Adaptors* are needed, as shown in Figure 5b. *Binning Adaptor* I is inserted into the launch flip-flop of the *Binning Critical Path* and *Binning Adaptor* II is inserted into the launch flip-flop of the upper stream path. The *Binning Adaptor* II keeps borrowing extra slack $S_{0}$ from the higher upper stream path ($P_{2}$), which ensures that the upper stream path ($P_{1}$) has an abundant margin to lend to the binning critical path. In addition, if the timing margin of both $P_{1}$ and $P_{2}$ is less than $S_{0}$, then $S_{0}$ should be reduced.

It should be noted that there could be more than one upper stream path ending at FF0 in Figure 2. Therefore, we should certify that the longest one has a slack larger than $S_{0}$.

### 2.3. Utilized Flash Memory

To permanently place the chip into the promoted bin, it is essential to store the Adapt_EN into non-volatile memory such as flash after the binning adaptation, as shown in Figure 1 and Figure 2. It needs to be noted that flash memory is not easy to be integrated in the same die with digital electronics. Considering that most chips have to load some configuration information at boot time, adaptation results (values of Adapt_EN) could be included in the configuration information, which is stored in external memory, such as flash. The *Binning Adaptor*s can read proper adaptation results from flash through on-chip registers after powering on or rebooting. The flash should be directly accessible by the proposed scheme. Therefore, the *Binning Adaptor* can read a proper adaptation signal directly from flash after powering on or rebooting. It should be noted that the utilized flash memory addresses should be writable only during the $F_{max}$ optimization process, which means the output of *Binning Checker* loses the control of *Binning Adaptor* after the optimization. In addition, if cost allows, the designer can integrate the one-time programmable (OTP) memory directly into the chip for Adapt_EN signals storage.

### 2.4. The Limitation and *Yield Optimization Rate* Estimation

**Figure 6:**
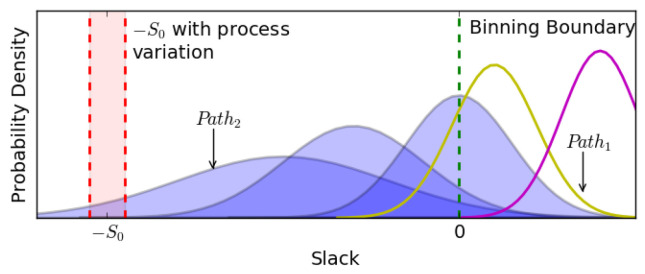
The *Binning Critical Path* selection and process variation affect the *Yield Optimization Rate*.

The bin promotion may not be successful for all devices. Figure 6 shows the silicon delay distributions of all speed-paths of a device, which can be binned at $F_{i}$. In other words, all speed-paths should have a probability of locating on the right side of the binning boundary. However, some Gaussian curves almost fully locate on the right side of the binning boundary, which represents the paths with little probability of causing binning failure at $F_{i}$. Meanwhile, the other Gaussian curves have a non-negligible part locating on the left side of the binning boundary, which represents the paths causing $F_{i}$ binning failure frequently. The shaded Gaussian curves in Figure 6 represent the selected *Binning Critical Paths*, and the $-S_{0}$ boundary marks the adaptability of *Binning Adaptor* with process variations.

Hence, there are two cases in which the bin promotion may not be achieved:

- Case 1: A chip can be promoted to the higher bin only if all silicon failure paths are successfully adapted. However, the selected *Binning Critical Paths* may not cover all silicon failure paths. If the delay of an unselected path exceeds the binning boundary on silicon, such as $Path_{1}$ in Figure 6, then the chip cannot be promoted to the higher bin.
- Case 2: Even if all silicon failure paths are selected as *Binning Critical Paths*, and equipped with *Binning Checker*s and *Adaptor*s, if the actual slack of a *Binning Critical Path* is smaller than $-S_{0}$, such as $Path_{2}$ in Figure 6, which means some failing paths are out of the adaptation range, then the bin promotion of the device also fails.

Hence, if we define the *Yield Optimization Rate* as the probability of successfully promoting a device to the higher bin, then it can be calculated as Equation (1), where *m* is the number of unselected paths with a probability of silicon failure belonging to Case 1, and *n* is the number of selected paths with a probability of being unadaptable belonging to Case 2. Here, *n* depends on the manufacturing technology, which is uncontrollable during the design stage. Reducing the number of *m* and adjusting $S_{0}$ are the best ways to improve the *Yield Optimization Rate*, which is introduced in Section 4.3.

(1) $$Yield\;Optimization\;Rate=\prod_{i=1}^{m}\int_{0}^{+\infty }p(t)dt\times \prod_{i=1}^{n}\int_{-S_{0}}^{0}p(t)dt$$

Note that process variations may affect the *Binning Checker*s and *Binning Adaptor*s. The impacts include (i) the scope of critical paths that the *Binning Checker* can recognize and (ii) the extra margin the *Binning Adaptor* can give. (i) and (ii) are designed to be the same as $S_{0}$; however, variations may make (i) and (ii) deviate from $S_{0}$. Hence, to reduce the impact of process variations, it is suggested to use the lowest variation rate (LVT large cell) to build the *Binning Checker*s and *Binning Adaptor*s. Section 4 provides the simulated and measured yield optimization results considering process variations. In Section 4, the measurement results shows that the binning promotion rate is 7–16%.

### 2.5. Application Scenarios

The proposed method is to reduce the impact of process variations on chip performance through post-manufacturing adaptation. During the design phase, the designer can optimize the timing of the circuit as much as possible. For example, by inserting registers, paths with large delay can be transformed into a two or multi-stage pipeline. However, due to design and optimization constraints, there will always be some paths that have greater delay than most paths, which are critical paths. These paths are the ones that need to be focused on. Even if the clock distribution tree is highly optimized, there may still be a sufficient margin in the upper stream path of the critical path.

To ensure that the critical path can run at the target frequency, if there is redundant timing in the clock tree, then we can adjust the clock tree so that the critical path can use the redundant timing of the clock distribution tree. This step can be performed during the design phase, or after being manufactured using the proposed method.

However, it is know that the process variation causes a gap between design and manufacturing. Even if the designer has highly optimized the timing at the design phase, the critical path or even non-critical path after being manufactured still has the probability of falling to a lower bin. If the designer improper utilizes redundant timing of the clock distribution tree at the design phase, it may make the upper stream paths of critical paths fail at the binning boundary after being manufactured, which means there are still limitations to timing optimization at the design phase.

For example, if the delay of a critical path is 980 ps, the delay of its upper stream path is 950 ps, the adaptable margin ($S_{0}$) is 10 ps, and the target clock period is 1000 ps. The adaptable margin is the margin that the upper stream path can lend to the critical path. Considering process variations, after being manufactured, the actual delay of the critical path may be 990 ps, the delay of its upper stream path may be 990 ps, and the adaptable margin ($S_{0}$) may be 11 ps. It is possible if the critical path is built with low variation cells (LVT large cells) for small delay, and the upper stream path is built with relatively higher variation cells (HVT small cells) for low area and low power overhead. If we perform timing adaptation at the design phase, both the critical path and its upper stream path could work at the target clock cycle during simulation. However, for the actual chip, the upper stream path would fail at the target clock period. However, if the designer adopts the proposed binning optimization method, he/she can perform adaptation according to the actual testing result. In this case, according to the test result, no critical path adjustment is needed, and both the critical path and its upper stream path could work at the target clock period. Hence, timing adaptation based on manufacturing test results can effectively improve the performance of the chip (speed bins).

In a digital circuit, optimizing and equalizing the pipeline stages is a common way to improve chip performance. Then, the upper stream path of the critical path may not have enough timing margin for adaptation. For this case, multiple *Binning Adaptor*s are needed, as shown in Figure 5b. It should be noted that the proposed method is not applicable if there are a large number of deep pipelines with close delay per stage in the circuit to be optimized.

In addition, there are also some circuits that use pipelines with as few stages as possible for low power consumption. There are some critical paths in such circuits that have a large impact on the circuit speed bins. In such design, retiming is limited because of the higher area and power consumption overhead it introduces. As a result, there will be some redundant timing of the clock distribution tree. At the same time, timing optimization during design stage, such as employing the redundant timing of the clock distribution tree, is impacted by the process variations as discussed above. In this case, using our proposed method, which adjusts the critical path clock after being manufactured, the performance of the chip can be improved with low overhead. The proposed *Binning Checker* only works during the speed binning test, which brings little power consumption.

As discussed above, considering the process variations, timing optimization using EDA tools during the design phase combined with timing adaptation using the proposed method after being manufactured is a better solution to achieve high chip performance.

It should be noted that there are limitations in the generality of the proposed adaptation methodology. In detail, the proposed methodology can be used to optimize the $F_{max}$ yield of a digital circuit when it has redundant timing in the clock tree, so that the critical paths can borrow sufficient timing margin from their upper stream paths for adaptation. If there is not enough timing margin for adaptation, multiple Binning Adaptors can be employed, as shown in Figure 5b. Our proposed methodology is not applicable if the circuit to be optimized has poor redundant timing in the clock tree, such as a circuit using a large number of deep pipelines with close delay per stage, which means that it would be hard for one stage to obtain sufficient timing margin from the previous stage.

## 3. The Flow for $F_{\max}$ Binning and Yield Optimization

**Figure 7:**
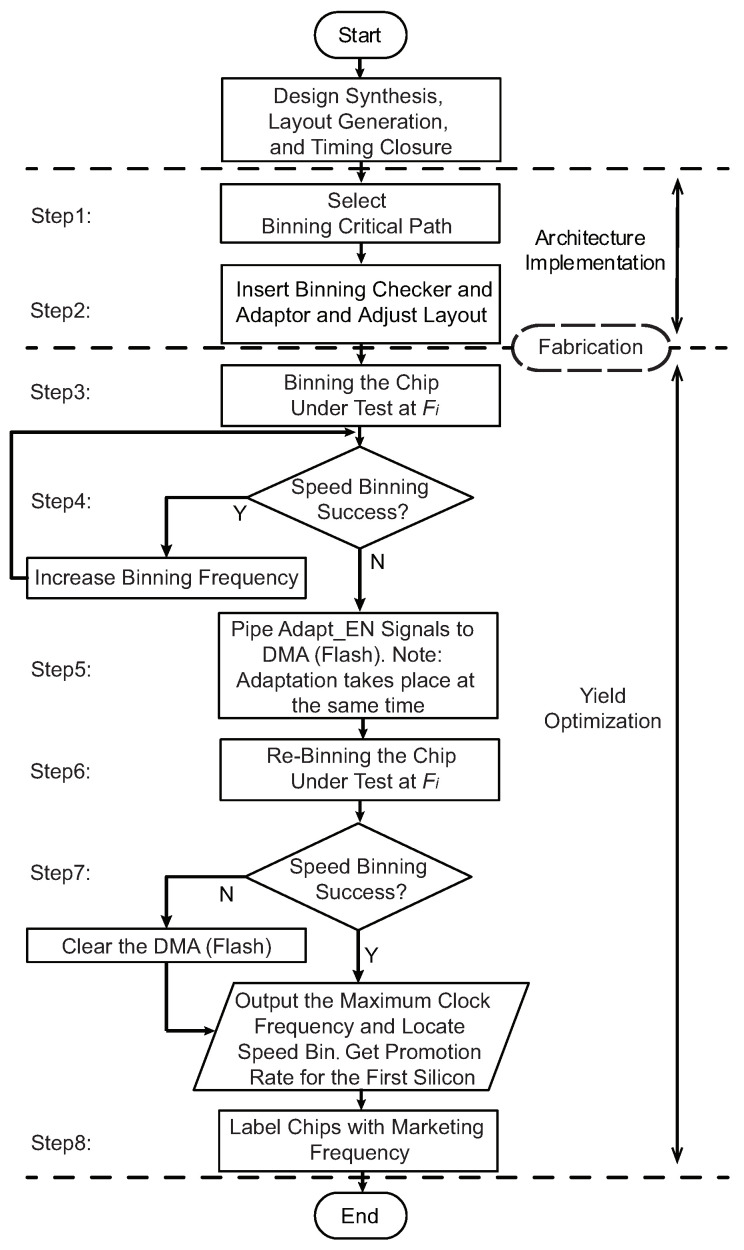
The proposed flow for binning and $F_{max}$ yield optimization.

The $F_{max}$ binning and yield optimization flow based on the adaptation system is shown in Figure 7.

***Step 1:*** Binning Critical Path Selection. As discussed above, the *Binning Critical Path* group size is limited by the acceptable overhead. However, to maximize the *Yield Optimization Rate*, the *Binning Critical Path* group should cover the paths causing speed binning failure with the highest probability. Therefore, designers should perform statistical timing analysis (STA) after layout generation and timing closure to select the most critical paths. The *Binning Critical Path* selection details and results for the implementation of this paper are shown in Section 4.3.

***Step 2:** Binning Checker* and *Binning Adaptor* Insertion. The *Binning Checker*s and *Binning Adaptor*s are inserted at the selected *Binning Critical Paths*. As discussed in Section 2, by replacing the clock buffers belonging to the original clock tree with the cells needed by the *Binning Adaptor*, the insertion process should not affect the already closed timing, and it requires minimum layout adjustments. The small overhead of the *Binning Checker* and *Binning Adaptor* helps to keep the overall area overhead of the adaptation system low.

***Step 3:*** Binning the Chip Under Test at $F_{i}$. In this step, the fabricated chip can be binned at frequency $F_{i}$ using functional, structural, or sensor-based speed binning methodologies. At the same time, the adaptable *Binning Critical Paths* are identified by the proposed system.

***Step 4:*** Get Preliminary Binning Result. In this step, if the chip under test passes the test at $F_{i}$, then the binning frequency is increased until reaching $F_{max}$. However, if the chip fails at $F_{i}$, the *Binning Checkers* identify adaptable *Binning Critical Paths*.

***Step 5:*** Perform Binning Adaptation. In this step, the Adapt_EN signals, which are the outputs of the *Binning Checkers*, are piped to a directly accessible non-volatile memory (DMA), and the binning adaptation is performed at the same time. The identified adaptable failing *Binning Critical Paths* in Step 4 are adapted.

***Step 6:*** Re-Binning at $F_{i}$. In this step, the chips under test are binned again at frequency $F_{i}$. If all of the silicon failure paths of a device have been adapted successfully, the re-binning passes. Hence, a percentage of the chips falling into the lower bin can be promoted to the higher bins. However, if the re-binning failed, the data in DMA should be cleared to ensure the chip still functions at the already passed lower bin.

***Step 7:*** Speed Bin Decision and *Yield Optimization Rate* Calculation. The speed bin of the chip under test can be decided according to whether the re-binning after adaptation is successful or not. By comparing the binning yield at Step 6 and Step 3, the *Yield Optimization Rate* can be obtained.

***Step 8:*** Label chips with Marketing Frequency. Aging and other factors would lead to performance degradation. Therefore, the marketing frequency of a device needs to add some additional margin based on the measured $F_{max}$.

As discussed above, the yield optimization flow can be integrated into all $F_{max}$ tests, such as the methods mentioned in [7,14], and the binning adaptation system brings little impact on the existing tests.

## 4. Experimental Results

The proposed on-chip binning and $F_{max}$ yield optimization system has been implemented onto several benchmarks from ITC’99, OpenSPARCT2 SoC, and ISCAS’89 benchmarks. The benchmarks equipped with the yield optimization system have been simulated in the 28 nm technology node [39] and implemented on a number of 28 nm Altera FPGAs.

### 4.1. The Verification of the *Binning Checker* and *Binning Adaptor*

**Figure 8:**
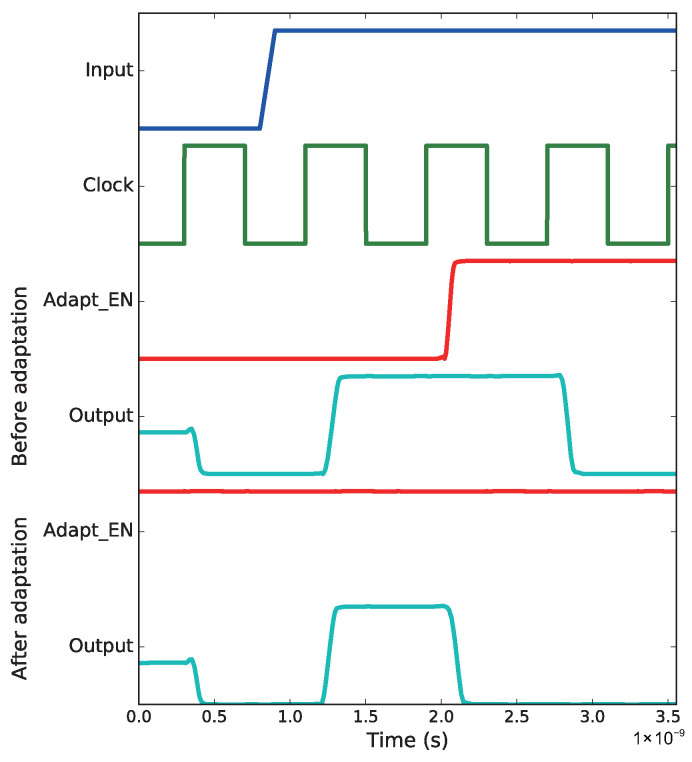
The Verification of *Binning Checker* and *Binning Adaptor*. Before adaptation, the *Binning Critical Path* fails at first capture, and it recovers at the second capture when the adaptation is enabled. After the adaptation status is kept into a directly accessible flash memory, the *Binning Critical Path* stays in the higher bin all the time.

To verify the proposed *Binning Checker* and *Binning Adaptor*, both are inserted into a b19 *Binning Critical Path* with a delay of 851 ps. The preset adaptable margin ($S_{0}$) is 23 ps, and the slack of the upper stream path is 50 ps. The Spice model of the *Binning Critical Path* together with the attached *Binning Checker* and *Binning Adaptor* are extracted from the design layout. A stimulus pattern with a rising edge is applied at the launch flip-flops of the critical path. The clock frequency is set to 1.19 GHz, and its period is smaller than the delay of the critical path. As shown in Figure 8, before adaptation, at the first capture, the path fails to make transition, which represents a 1.19 GHz binning failure. At the same time, the *Binning Checker* identifies that the monitored path is an adaptable *Binning Critical Path*, and it switches *Adapt_EN* to *1*, which initiates the adaptation. Thus, the same path functions again at the second capture, which means the path meets the 1.19 GHz binning frequency. The waveforms after adaptation in Figure 8 represent the case when the adaptation status of the specific path has been written into a directly accessible flash memory and applied to the path right after power up or rebooting. Then, when the same patterns are re-applied to path, the path always functions correctly at 1.19 GHz frequency.

### 4.2. A Successful Bin Promotion Case

For the ITC’99 b19 benchmark, through using statistical statistic timing analysis, a total of 120 top paths are selected as the *Binning Critical Paths*. According to the design of the *Binning Checker* and *Binning Adaptor*, the adaptable margin $S_{0}$ equals 0.3 ns. Then, the speed bin of a b19 circuit is simulated by HSpice with and without binning adaptation.

**Figure 9:**
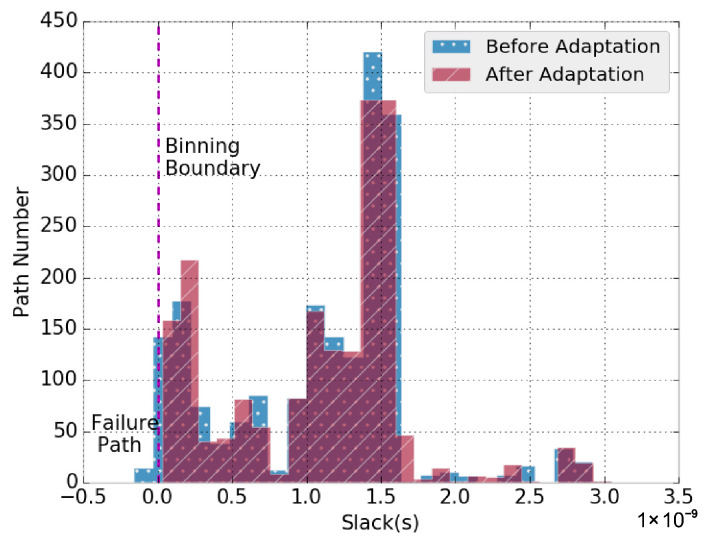
The slack distribution of a ITC’99 b19 sample, with 94 paths fail 167 MHz binning. With all failure paths correctly adapted, this sample is successfully promoted to the 167 MHz bin.

As shown in Figure 9, the *x*-axis shows the slack of *Binning Critical Paths*, where $F_{i}$ is the binning frequency equals to 167 MHz. Hence, the vertical line at *0* represents the binning boundary. The path slack distribution before and after binning adaptation are shown as the dotted and shaded bars, respectively. It can be seen that without binning adaptation, 94 paths fall on the left side of the binning boundary, which means they cause the binning failure of the chip at 167 MHz. However, the worst slack of the failure path is 0.16 ns, which is within the adaptable margin $S_{0}$ = 0.3 ns. Hence, with *Binning Checker* and *Binning Adaptor* inserted on the 120 *Binning Critical Paths*, which cover all 94 failing paths, after adaptation, the slack of all paths are re-distributed on the right side of the binning boundary (Figure 9), which means that this chip is successfully promoted to the 167 MHz bin. It should be noted that as discussed above, depending on the *Binning Critical Path* and adaptable margin $S_{0}$ selection, successful binning promotion may not always be possible. Section 4.3 discusses the impact of the above two factors on the number of devices promoted to the higher bin (*Yield Optimization Rate*).

### 4.3. The Selection of *Binning Critical Path* and Adaptable Margin $S_{0}$

**Figure 10:**
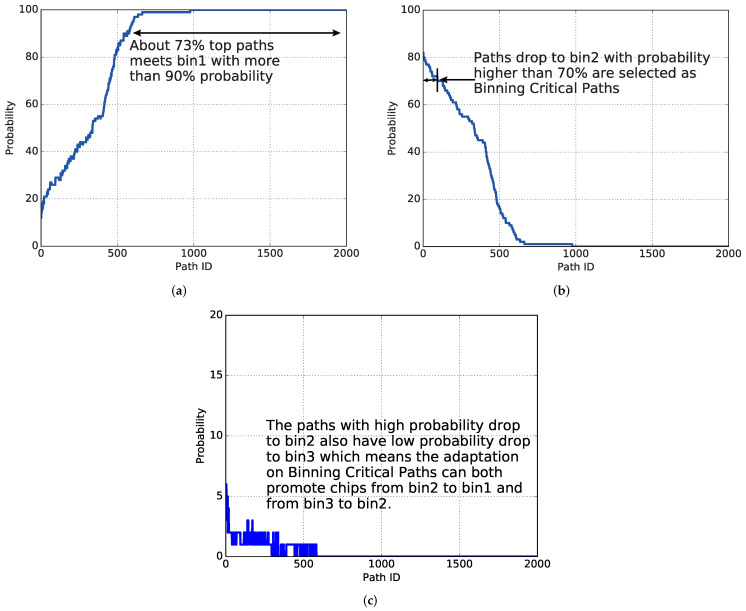
For the first silicon, the *Binning Critical Paths* are selected based on statistical timing analysis. The paths with high probability (δ = 70% in this implementation) falling into the lower bins are selected as *Binning Critical Paths*. (**a**) The probability of ITC’99 b19 top 20% paths locate in bin1; (**b**) The probability of ITC’99 b19 top 20% paths locate in bin2; (**c**) The probability of ITC’99 b19 top 20% paths locate in bin3.

According to Equation (1), to maximize the *Yield Optimization Rate*, for a given *Binning Critical Path* group size (overhead), the silicon failure path coverage should be maximized. Hence, for the first silicon, when no silicon data are available, the designer needs to perform Monte Carlo analysis to obtain the probability of each path to locate in a certain bin. Figure 10 shows the probability of b19’s speed paths (top 20% paths) located in bin1 (167 MHz), bin2 (100 MHz), and bin 3 (33 MHz) (considering 10% L, Nmos: vth0 = 17% toxref = 10%; Pmos: vth0 = 18% toxref = 10% process variations, 1000 Monte Carlo simulations). It can be seen that as shown in Figure 10a, about 73% of the speed paths locate in bin1 with more than 90% probability, and 5% of the speed paths drop to bin2 with more than 70% probability, as shown in Figure 10b. If the *Binning Critical Path* selection criteria δ is set as 70%, then the 5% of speed paths with more than δ probability dropping to bin2 (marked in Figure 10b) are selected as *Binning Critical Paths*. By comparing the curves in Figure 10b,c, the paths with higher probability of dropping to bin2 are the same group of paths with a lower probability to drop to bin3. Therefore, the same adaptation architectures help to promote chips from bin2 to bin1 as well as from bin3 to bin2.

**Figure 11:**
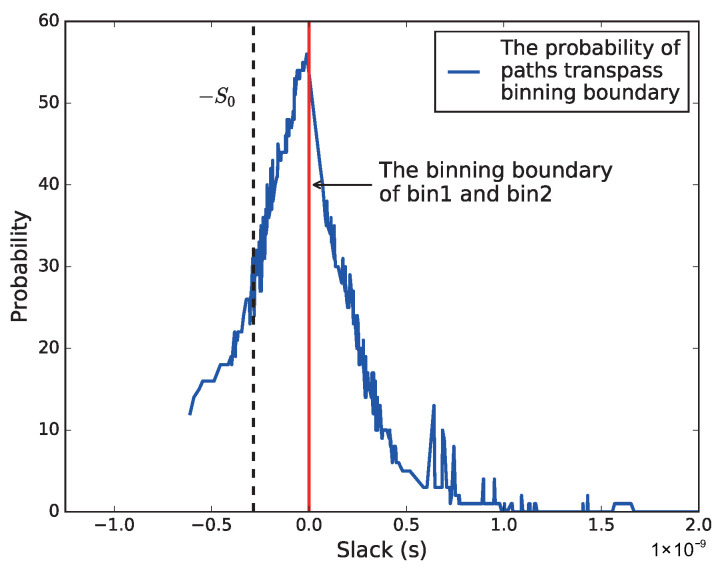
The probability of paths transpassing bin boundaries with different slack. Adaptable margin $S_{0}$ is selected according to δ (70% in this implementation).

Figure 11 shows the average slack of the b19’s top 20% paths at $F_{i}$ and their corresponding probability of transpassing the bin boundary. For example, for the paths with average slack smaller than 0, the *y*-axis represents the probability of the path locating in the higher bin ($F_{i}$). While for the paths with average slack larger than 0, the *y*-axis represents the probability of the path falling to the lower bin. Hence, if we make the *Binning Critical Path* selection criteria δ as 70% (with a probability of δ dropping to the lower bin, and a probability of 1−δ = 30% staying in the $F_{i}$ bin), then the ideal adaptable margin $S_{0}$ can be decided, as shown in Figure 11. Hence, the BUFF0 and BUFF2 inside the *Binning Checker* and *Binning Adaptor* in Figure 2 can be selected accordingly.

There is no doubt that the more paths that are selected as *Binning Critical Paths* with *Binning Checker* and *Binning Adaptor* inserted, the more chips can be promoted to higher bins. However, redundant *Binning Checker* and *Binning Adaptor* can bring higher overhead and may be not necessary. The ideal condition is that all paths drop out of bin $F_{i}$, and no paths within $F_{i}$ are selected as *Binning Critical Paths*. However, this cannot be guaranteed based on the statistical results shown in Figure 10. Instead, the paths with a probability larger than δ (the *Binning Critical Paths* selection criteria) are selected to compose the *Binning Critical Paths* group.

**Figure 12:**
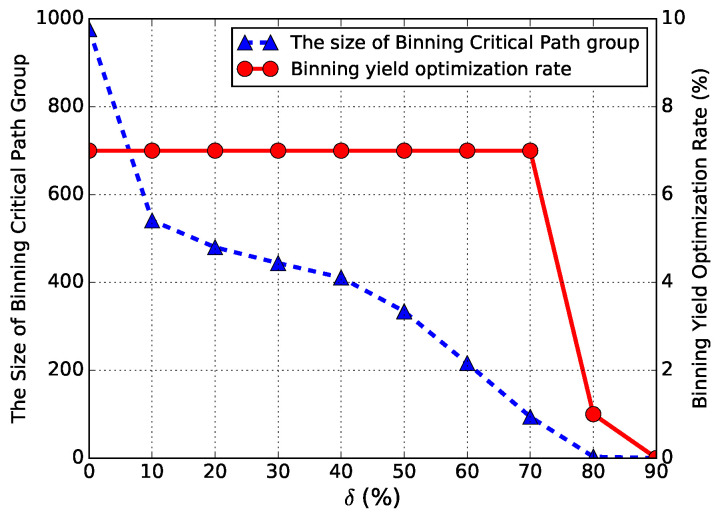
The impact of *Binning Critical Path* selection criteria (δ) on the size of *Binning Critical Path* group, as well as the *Yield Optimization Rate*, considering 10% L, Nmos: vth0 = 17% toxref = 10%; Pmos: vth0 = 18% toxref = 10% process variations for b19.

Figure 12 shows the impact of δ on the size of *Binning Critical Paths* group, as well as the *Yield Optimization Rate* (the percentage of devices being promoted to the higher bin), for b19. It can be seen that as δ decreases, the *Binning Critical Path* group size is increasing. However the *Yield Optimization Rate* almost remains stable after δ reaches 70%. The reason is, according to Figure 11, with fixed $S_{0}$, even more paths are selected as *Binning Critical Paths* by decreasing the selection criteria δ, the newly selected paths are with slacks out of the adaptable margin ($S_{0}$). Therefore, over increasing the *Binning Critical Path* group size gives little help for yield optimization. It should be noted that different devices have different δ and $S_{0}$, but the method to find them is the same.

**Figure 13:**
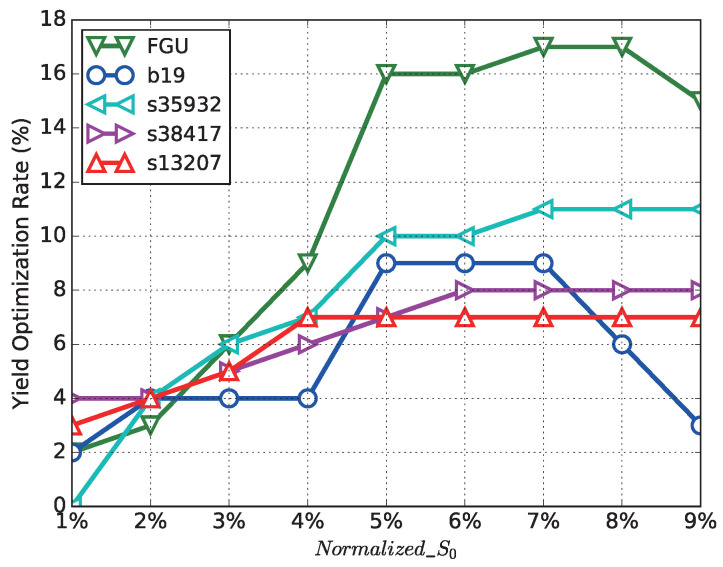
The influence of $Normalized\_S_{0}$ on *Yield Optimization Rate* for different benchmarks.

As discussed above, the decision of adaptable margin $S_{0}$, which is the timing margin borrowed from the upper stream path of a timing critical path, also affects the *Yield Optimization Rate*. To analyze $S_{0}$’s impact, we introduce $Normalized\_S_{0}$ defined in Formula (2), where $T_{sys}$ is the functional clock cycle length. It seems that the larger the $Normalized\_S_{0}$ is, the more binning failure paths can be recovered. However, Figure 13 shows the *Yield Optimization Rate* through increasing $Normalized\_S_{0}$. It can be seen that at the beginning, with the increment of $Normalized\_S_{0}$, the *Yield Optimization Rate* improves. However, as the timing margin of upper stream path is restricted, $S_{0}$ cannot keep increasing. A too-aggressive $S_{0}$ fails the upper stream path. Take b19 for example, as shown in the same figure, with $Normalized\_S_{0}$ increased to 6%, the *Yield Optimization Rate* starts to decrease. Therefore, for this implementation, it is suitable to set the value of $Normalized\_S_{0}$ to 5%.

(2) $$Normalized\_S_{0}=\frac{S_{0}}{T_{sys}}$$

Due to the existing of the difference between manufactural and statistical simulated path delay, the actual *Binning Critical Paths* might be different from the simulated ones. Therefore, to further improve the bin promotion rate, it is suggested to improve the timing library according to the silicon data.

### 4.4. The Profit Increment Due to Yield Optimization

**Figure 14:**
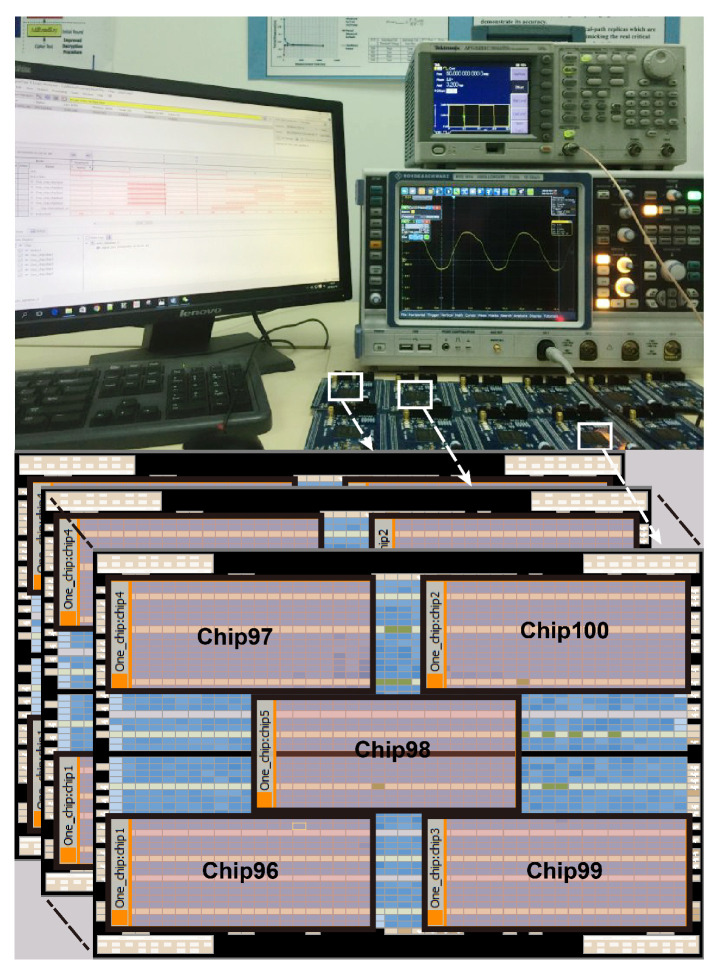
Overview of 28 nm FPGA (Altera 5CEFA4F23C8N) chip planner and measurement setup.

To mimic the actual speed binning scenario, the proposed binning adaptation system has been implemented on a number of FPGAs. The technology of the adopted FPGA is 28 nm, which has enough process variations. Each FPGA represents one or multiple benchmarks depending on the size of the implemented benchmark. The layout and measurement setup of FPGAs are shown in Figure 14. The chips will be projected into three bins according to their $F_{max}$ test results.

**Figure 15:**
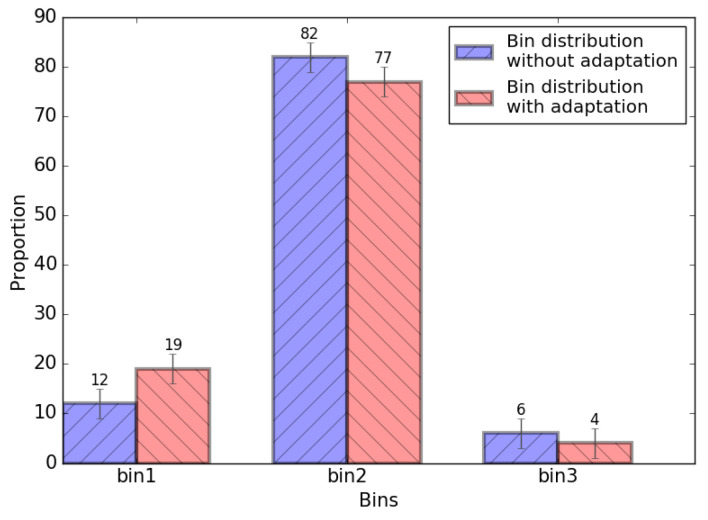
The measured *Yield Optimization Rate* for b19.

For b19, 120 paths are selected as the binning critical path and inserted with *Binning Checker*s and *Binning Adaptor*s, according to Figure 10. The distribution of 100 b19 chips before and after binning adaptation is shown in Figure 15. It can be seen that considering the 100 b19 FPGA samples, for the first silicon, two chips are promoted from bin3 into bin2, and seven chips are promoted from bin2 into bin1. Therefore, for the first silicon, 9% chips are upgraded to higher bins with the proposed binning adaptation system, with *Yield Optimization Rate* equaling to 9%.

It is known that the profit of higher bin chips is more than that of the lower bins chips. For instance, the price of higher bin Altera FPGA is about 1.2 times of the next bin [40]. This would help to evaluate the Value-Added Profits (*VAP*). *VAP* is defined by Formula (3), where $Num\_after_{bin[i]}$ and $Num\_beforer_{bin[i]}$ represent the number of chip fall in bin[i] after and before adaptation, respectively. α is the price of higher bin devices over the lower bin, and *k* is the number of bins.

(3) $$VAP=\frac{\sum_{i=1}^{k}Num\_after_{bin[i]}\alpha^{k-i}}{\sum_{k=1}^{\alpha }Num\_before_{bin[i]}\alpha^{k-i}}-1$$

As α = 1.2, and *k* = 3 for this implementation, the *Yield Optimization Rate* and Value-Added Profit results for different benchmarks are shown in Table 1. From the table, it can be seen that under the given value of *Binning Critical Path* selection criteria δ and adaptable margin $S_{0}$, the *Yield Optimization Rate* is 7–16% and the *VAP* is 1.18–3.04%,

### 4.5. The Area Overhead

Table 2 shows the number of *Binning Checker* and *Binning Adaptor* pairs inserted into different benchmarks and the overall area overhead of the proposed system. It can been seen that the proposed system has a low area overhead. Note that the overheads can be even smaller for a large-scale industrial application.

## 5. Conclusions

In this paper, a novel on-chip adaptation-based $F_{max}$ binning and optimization methodology is proposed, which can upgrade marginal chips and improve the yield of high-performance chips. The proposed adaptive system has been implemented and validated on five benchmarks from ITC, ISCAS89, and OpenSPARCT2 core. In addition, it has been verified on a number of FPGAs. The experiment result shows that the proposed $F_{max}$ binning and optimization methodology can improve the yield of high-end chips up to 7–16%, and improve the manufacturer’s profit by 1.18–3.04% according to the *VAP* model. At the same time, the all-digital adaptation system brings low design overhead and low area overhead.

## Figures and Tables

**Table 1:** The *Yield Optimization Rate* and Value-Added Profit (*VAP*) with the proposed adaptation on various benchmarks.

Benchmark	s13207	s38417	s35932	b19	FGU
*Yield Optimization Rate*	7%	8%	10%	9%	16%
Value-added Profit (*VAP*)	1.18%	1.34%	1.85%	1.71%	3.04%

**Table 2:** The area overhead of the proposed system on different benchmarks.

Benchmark	s13207	s38417	s35932	b19	FGU
Pairs of Checker and Adaptor	6	9	43	120	84
Total Area (μm^2^)	11,249.55	35,740.03	38,923.68	412,647.52	1,246,460.42
Overall Area Overhead	1.55%	0.73%	3.20%	0.85%	0.19%
Required DMA Bit Number	6	9	43	120	84

## Data Availability

Not applicable.
